# Utility of Platelet Indices as Prognostic Markers of Sepsis: A Medical Intensive Care Unit-Based Cross-Sectional Study at a Rural Setup

**DOI:** 10.7759/cureus.54490

**Published:** 2024-02-19

**Authors:** Anusha Gupta, Sunil Kumar, Sourya Acharya, Rajesh Sarode, Sachin Agrawal, Rinkle Gemnani, Keyur Saboo, Nikhil Reddy

**Affiliations:** 1 Department of Gastroenterology, Jawaharlal Nehru Medical College, Datta Meghe Institute of Higher Education & Research, Wardha, IND; 2 Department of Medicine, Jawaharlal Nehru Medical College, Datta Meghe Institute of Higher Education & Research, Wardha, IND

**Keywords:** plateletcrit, platelet distribution width, mean platelet volume, intensive care unit, mortality, sepsis

## Abstract

Introduction

Even after the breakthrough advancements in the management and prognostic scoring of sepsis, it remains an important cause of morbidity and mortality encountered in intensive care units (ICUs) throughout the globe. This study highlights the utility of platelet indices as prognostic markers of sepsis.

Methods

In the present prospective cross-sectional study, a total of 177 patients with sepsis were enrolled using the Sepsis-3 criteria. The platelet indices were then linked to severity using the Acute Physiology and Chronic Health Evaluation (APACHE) II score. The correlation of platelet indices to morbidity in terms of the length of ICU stay, need for a mechanical ventilator, types of infection, and mortality was also assessed.

Results

The results showed that mean platelet volume (p = 0.004) and platelet distribution width (PDW; p = 0.009) were positively correlated with the severity of sepsis. Among all the parameters, plateletcrit (%) was the best predictor of the need for an invasive mechanical ventilator at a cutoff point of ≤0.22 with a 60.90% chance of correctly predicting the need for an invasive mechanical ventilator, as was mortality at a cutoff point of ≤0.22 with a 67.30% chance of correct prediction. Among the platelet indices, only PDW showed a significant association with growth in culture because patients with growth had significantly higher PDW as compared to those who did not have growth (22.4 ± 4.47 vs 20.81 ± 4.29, p = 0.011).

Conclusion

The difference between the survivors and non-survivor groups was statistically significant for platelet indices, making them easily available, cost-effective, and useful prognostic markers for patients in septic shock. This will help in easy understanding and preventing its morbid complications, even at the primary care physician level.

## Introduction

In the critically ill, sepsis and its complications remain one of the major causes of morbidity and mortality [1]. Infection, whether reported or suspected, and some of the signs and symptoms of an inflammatory reaction (in adults), are found in sepsis [2,3]. The causes of sepsis are multifactorial, but virtually every infectious organism can be involved. Bacterial Gram-negative infections are far more common as a cause of sepsis syndromes, with a frequency of 62%, followed by Gram-positive infections at 47%, according to the 2009 Extended Prevalence of Infection in Intensive Care (EPIC II) study [4].

Sepsis can begin anywhere inside the body with a source of infection - the urinary tract, lungs, skin, peritoneal cavity, and others. As microbial components are recognized by special pattern recognition molecules (CD14 cells and Toll-like receptors), a complex cellular activation process follows, consisting of cytokine release; neutrophil, monocyte, and endothelial cell activation; neuroendocrine involvement; and complement, coagulation, and fibrinolytic system activation [5].

Mean platelet volume (MPV) is a platelet index that has been available since the 1970s [6]. Other platelet indices, including platelet distribution width (PDW) and plateletcrit (PCT), have been added since then. Evidence of MPV changes in sepsis is currently controversial: some studies suggest that MPV increases during severe sepsis, whereas others show a decrease [7].

When turnover is accelerated, the PDW increases during platelet depletion and shares similar activity with MPV during acute infections. PCT is the platelet index that is affected by platelet number and size and has a positive relationship with the number of platelets [8].

Various studies have evaluated different scoring systems as well as music therapy regarding the outcomes of critically ill patients admitted to the medical intensive care unit (MICU) [7-10]. A study by Jawaharani et al. has shown that music therapy has a better outcome in critically ill patients with sepsis [11]. One study studied red cell distribution width and PDW as significant indicators for the period of stay in the MICU, the requirement for mechanical ventilation, and mortality [10]. There are few studies conducted in India that have assessed the role of platelet indices in sepsis [12].

This study had been planned to assess the impact of platelet indices as prognostic markers of sepsis-like morbidity, ICU stay, mortality, and the cause of sepsis (Gram-positive or Gram-negative infections).

## Materials and methods

This prospective cross-sectional study was conducted in the MICU under the Department of Medicine at Jawaharlal Nehru Medical College, Wardha, India, from October 2018 to July 2020 after clearance from the Institutional Ethical Committee (approval number DMIMS (DU)/IEC/2018-19/7549). All the patients, regardless of gender and above 18 years of age, who were admitted to the MICU with a clear diagnosis of sepsis based on Sepsis-3 criteria, were included in the study. Patients transferred to the MICU from outside the hospital, patients with clear no sepsis diagnoses (e.g., trauma, myocardial infarction, and pulmonary embolism), post-surgical patients, and patients not willing to give informed consent were excluded from the study. The Sepsis-3 criteria include two components: “Suspected (or documented) infection and an acute increase in ≥2 sepsis-related organ failure assessment (SOFA) points” and “Suspected (or documented) infection plus vasopressor therapy needed to maintain mean arterial pressure at ≥65 mmHg and serum lactate >2.0 mmol/L despite adequate fluid resuscitation.” The SOFA score is a 24-point measure of organ dysfunction, assessing six organ systems (renal, cardiovascular, pulmonary, hepatic, neurologic, and hematologic), with 0-4 points assigned per organ system [6].

A detailed clinical examination was also done. A complete blood count was done after withdrawing samples aseptically at the time of admission. A repeat sample was not taken into consideration. The sample was collected in a dipotassium ethylenediamine tetraacetic acid (EDTA) bulb and tested within one hour when maintained at room temperature. In the case of a delay, the samples were cooled in a refrigerator until they were processed. An automated cell counter, the Beckman Coulter Unicel DxH 800 Hematology Analyzer (Beckman Coulter, Inc., Brea, CA, USA), was used, which provided the values of hemoglobin, total platelet count, and total leukocyte count. Blood was drawn aseptically from vacuumed plain bulbs that contained clot activators. Platelet indices were obtained from the Complete Blood Count Coulter report (three parts), parameters needed for the calculation of the Acute Physiology and Chronic Health Evaluation (APACHE) II scores were taken, and the calculation of the APACHE II score was done using a software-based online module. The correlation of platelet indices such as MPV, PDW, and PCT with outcomes such as the use of mechanical ventilators, length of ICU stays, and discharge and death was done. The platelet indices were also correlated with the type of infection causing sepsis. Blood cultures were collected from the patients and sent for Gram staining. The peripheral blood samples from the cases were collected in EDTA vials.

Statistical analysis

The data were coded and recorded in Microsoft Excel (Microsoft Corporation, Redmond, WA, USA). Medians and means were used to elaborate the descriptive statistics, and IQRs and SDs for variables that were continuous and for variables that were categorical percentages and frequencies were used. Differences in the group for categorical data were compared with the help of the chi-squared test or Fisher’s exact test. Statistical analysis was carried out using IBM SPSS Statistics for Windows, Version 23 (Released 2015; IBM Corp., Armonk, NY, USA). Statistical significance was set at p < 0.05.

## Results

Out of 177 patients, the mean age was 51.9 ± 18.2 years, of which 57% were males. Platelet indices were MPV (femtoliter) 11.43 ± 1.72, PCT (%) 0.5 ± 0.52, and PDW (%) 21.45 ± 4.42 in all consecutive patients. Other baseline characteristics are shown in Table 1.

**Table 1:** Baseline characteristics of the patients APACHE, Acute Physiology and Chronic Health Evaluation; fL, femtoliter; ICU, intensive care unit; MPV, mean platelet volume; PCT, plateletcrit; PDW, platelet distribution width

All parameters	Number (%)
Age (years)	Mean ± SD (51.9 ± 18.2)
16-40 years	50 (28.25%)
40-60 years	74 (41.81%)
>60 years	53 (29.94%)
Gender	
Male	102 (57.63%)
Female	75 (42.37%)
Outcome	
Discharge	118 (66.67%)
Death	59 (33.33%)
APACHE II score	28.08 ± 11.6
White blood cells (cells/cumm)	19,129.49 ± 10,587.63
Length of ICU stay (days)	8.75 ± 4.55
Mechanical ventilator	
Not required	101 (57.06%)
Required	76 (42.94%)
Platelet indices	
Total platelet count (per cumm)	1.38 ± 0.88
MPV (fL)	11.43 ± 1.72
PCT (%)	0.5 ± 0.52
PDW (%)	21.45 ± 4.42
Growth in culture	
Growth seen	71 (40.11%)
No growth	106 (59.89%)
Type of growth in culture	
Gram-positive	18 (25.35%)
Gram-negative	50 (70.42%)
Others	3 (4.23%)

**Figure 1:**
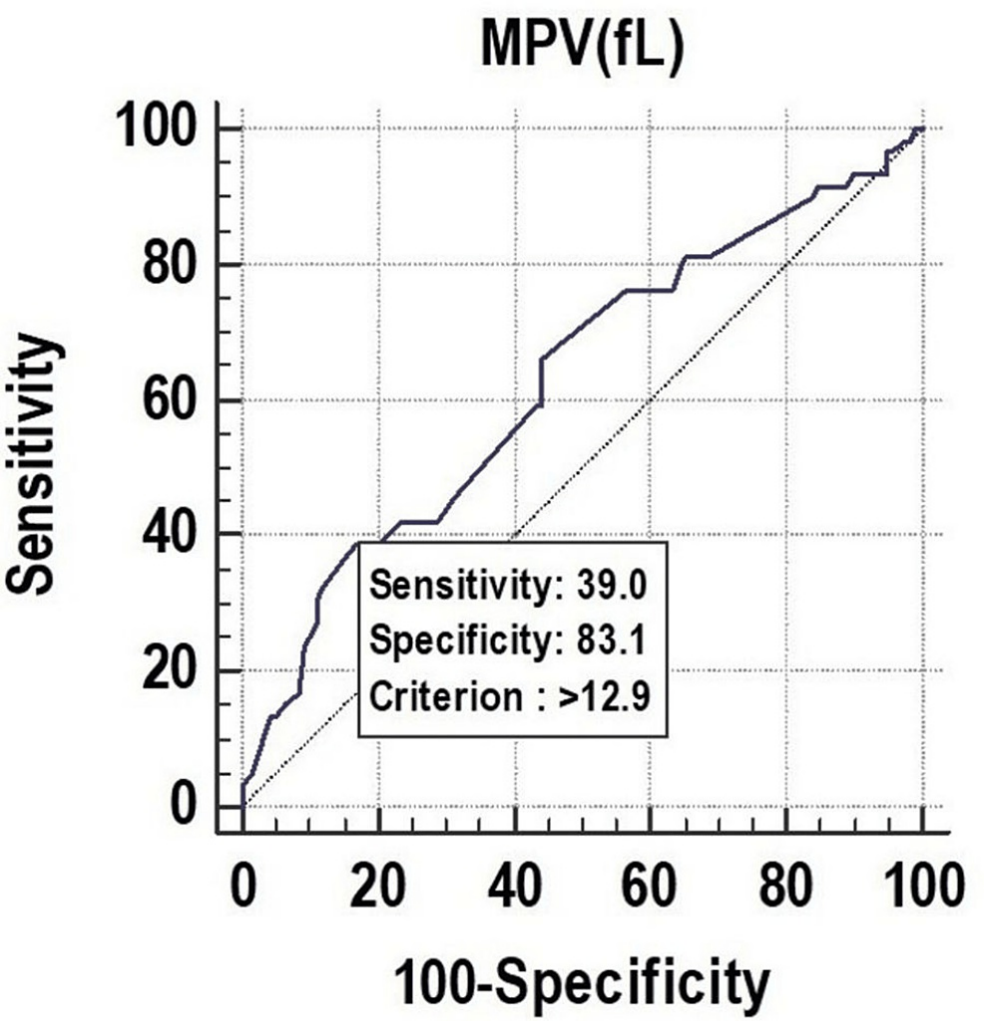
Receiver operating characteristics of MPV (fL) for predicting mortality fL, femtoliter; MPV, mean platelet volume

**Figure 2:**
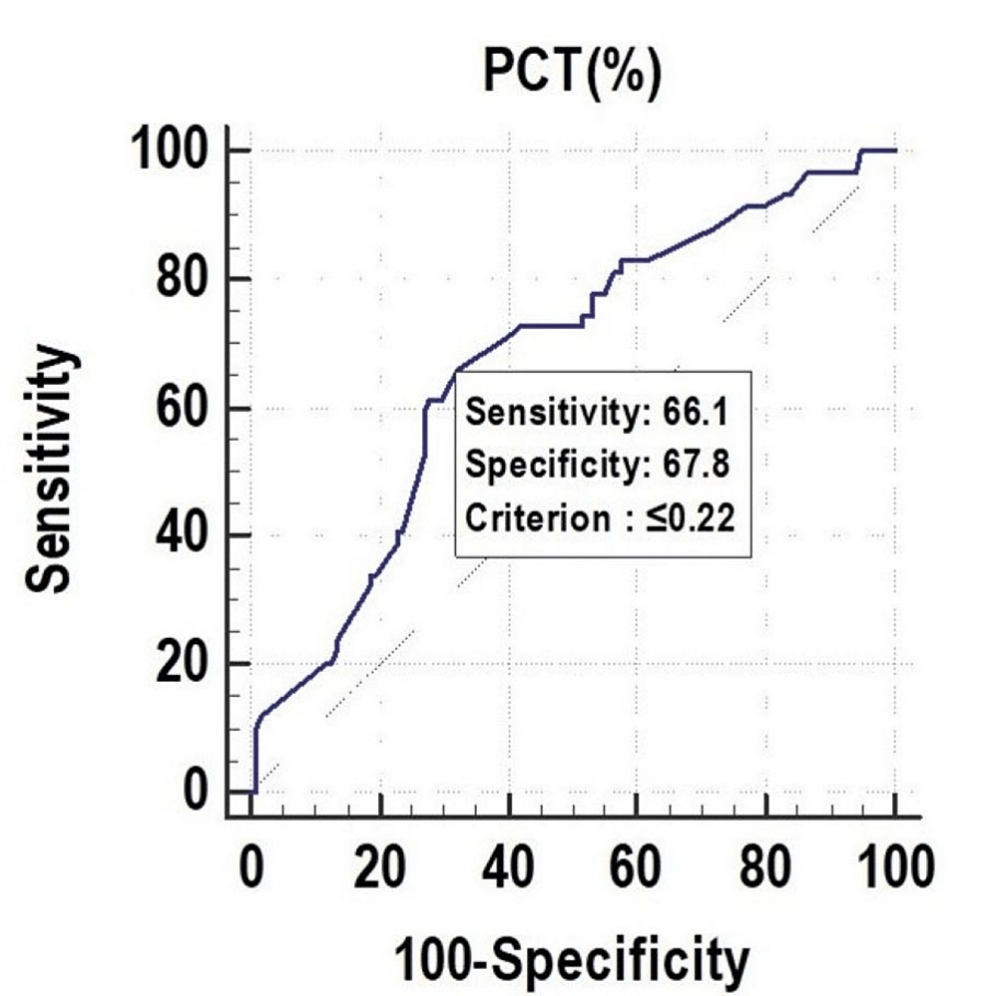
Receiver operating characteristics of PCT (%) for predicting mortality PCT, plateletcrit

**Figure 3:**
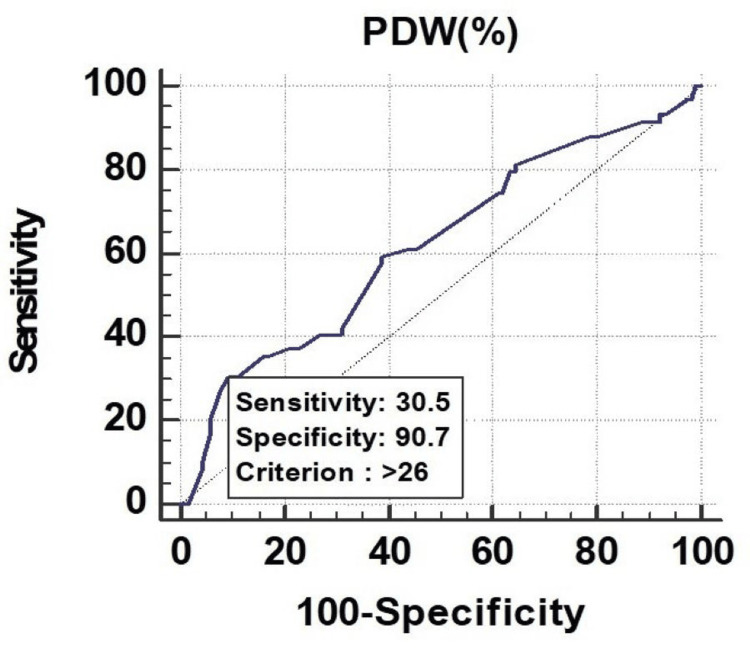
Receiver operating characteristics of PDW (%) for predicting mortality PDW, platelet distribution width

All the parameters had significant discriminatory power to predict mortality. The discriminatory power of MPV (fL) (AUC: 0.629; 95% CI: 0.553-0.700), PCT (%) (AUC: 0.673; 95% CI: 0.599-0.742), and PDW (%) (AUC: 0.619; 95% CI: 0.543-0.691) was acceptable. Among all the parameters, PCT (%) was the best predictor of mortality at a cutoff point of ≤0.22, with a 67.30% chance of correctly predicting mortality, as shown in Figure 1, Figure 2, and Figure 3.

**Figure 4:**
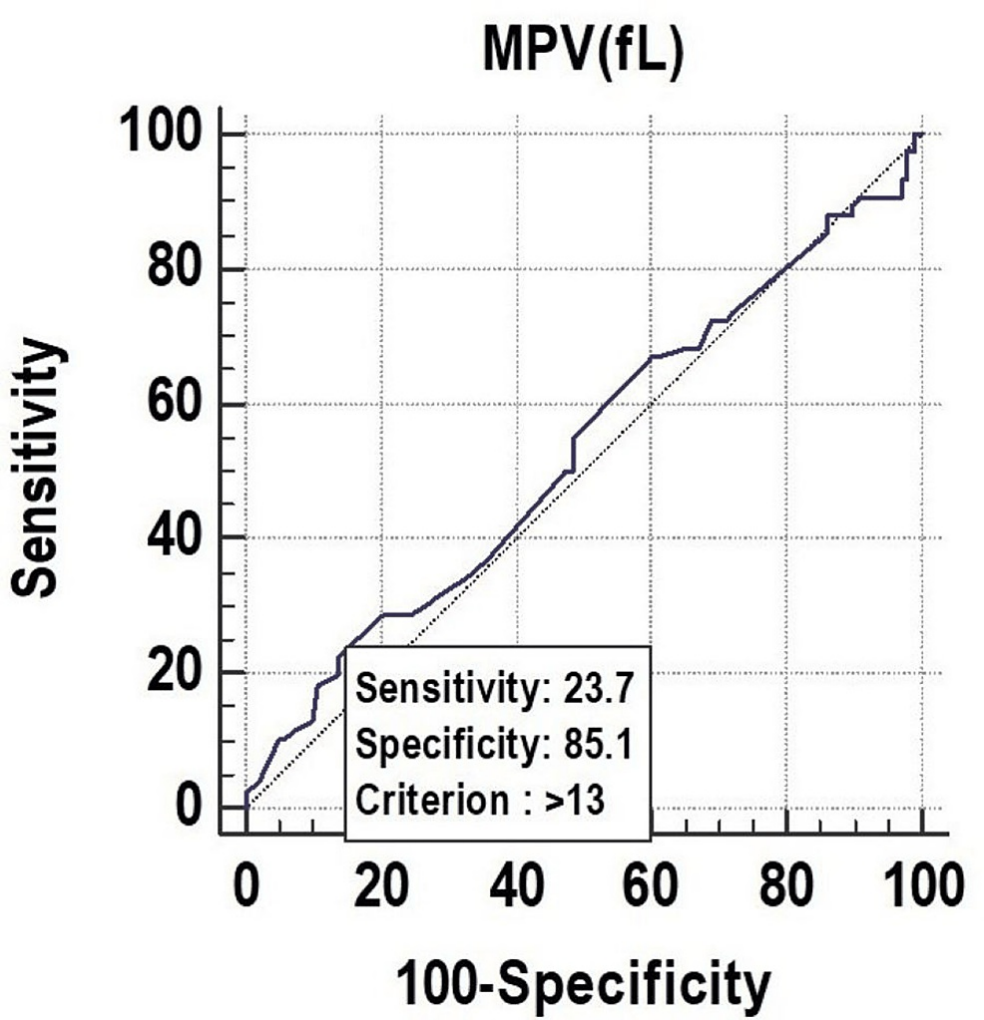
Receiver operating characteristics of MPV (fL) for predicting the need for a ventilator fL, femtoliter; MPV, mean platelet volume

**Figure 5:**
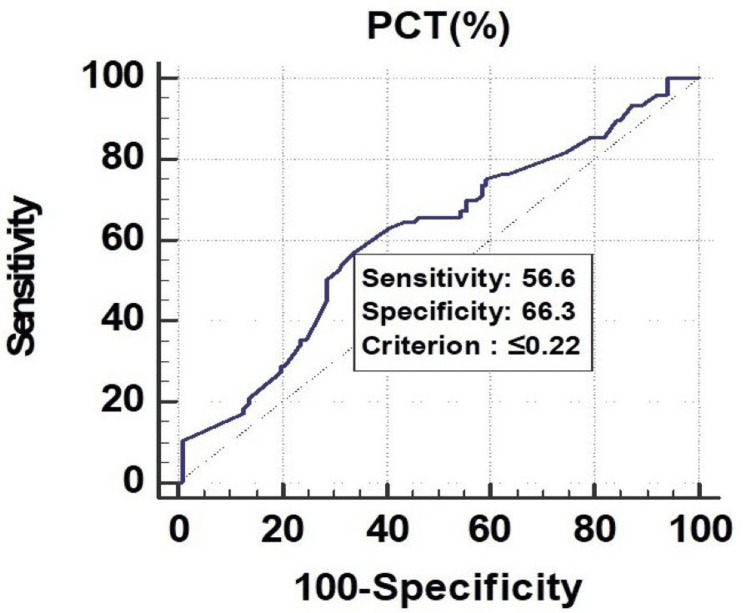
Receiver operating characteristics of PCT (%) for predicting the need for a ventilator PCT, plateletcrit

**Figure 6:**
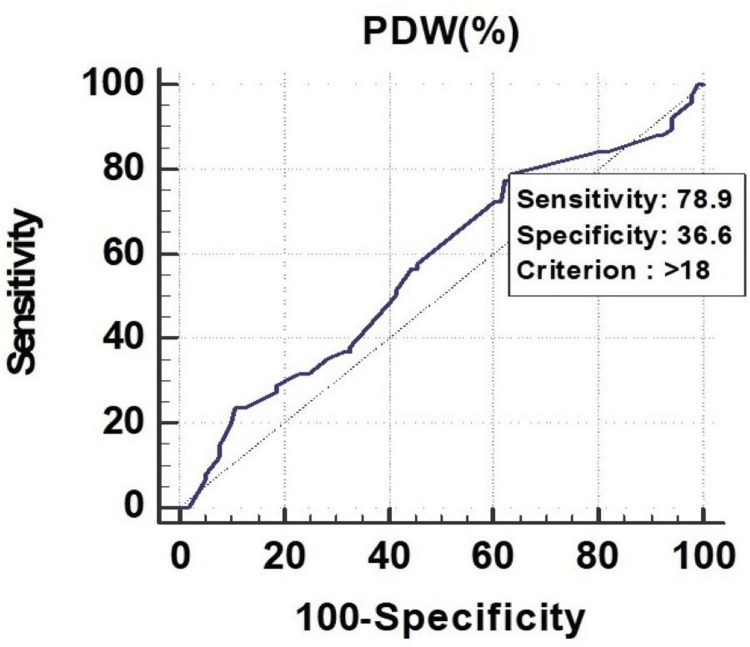
Receiver operating characteristics of PDW (%) for predicting the need for a ventilator PDW, platelet distribution width

Receiver operating characteristic curves above the diagonal line are considered to have a reasonable discriminating ability to predict the need for a ventilator. The discriminatory power of PCT (%) (AUC: 0.609; 95% CI: 0.533-0.682) was acceptable. On the other hand, the discriminatory power of MPV (fL) (AUC: 0.529; 95% CI: 0.453-0.605) and PDW (%) (AUC: 0.569; 95% CI: 0.493-0.643) was nonsignificant. Among all the parameters, PCT (%) was the best predictor of the need for a ventilator at the cutoff point of ≤0.22, with a 60.90% chance of correctly predicting the need for ventilators in Figure 4, Figure 5, and Figure 6.

PCT was statistically significant in the case of the need for ventilators (0.39 ± 0.47; p = 0.012), as shown in Table 2. PDW (%), PCT (%), and MPV (fL) were statistically significant (22.73 ± 4.74; 0.3 ± 0.42; 11.92 ± 1.76, p = 0.009, 0.0002, and 0.005), respectively, in terms of mortality as shown in Table 3. The association of platelet parameters with length of ICU stay has been highlighted in Table 4.

**Table 2:** Association of MPV (fL), PDW (%), and PCT (%) with the need for a ventilator fL, femtoliter; MPV, mean platelet volume; PCT, plateletcrit; PDW, platelet distribution width

	No need for a mechanical ventilator (n = 101)	Need for a mechanical ventilator (n = 76)	Total	p-value	Mann-Whitney test
MPV (fL) mean ± SD	11.37 ± 1.66	11.51 ± 1.82	11.43 ± 1.72	0.501	3,612
PDW (%) mean ± SD	21.01 ± 4.28	22.02 ± 4.56	21.45 ± 4.42	0.114	3,306.5
PCT (%) mean ± SD	0.59 ± 0.54	0.39 ± 0.47	0.5 ± 0.52	0.012	2,998

**Table 3:** Association of PDW (%), PCT (%), and MPV (fL) with mortality fL, femtoliter; MPV, mean platelet volume; PCT, plateletcrit; PDW, platelet distribution width

	Death (n = 59)	Discharge (n = 118)	Total	p-value	Mann-Whitney test
PDW (%) mean ± SD	22.73 ± 4.74	20.81 ± 4.12	21.45 ± 4.42	0.009	2,654.5
PCT (%) mean ± SD	0.3 ± 0.42	0.6 ± 0.53	0.5 ± 0.52	0.0002	2,274
MPV (fL) mean ± SD	11.92 ± 1.76	11.19 ± 1.66	11.43 ± 1.72	0.005	2,585

**Table 4:** Association of PDW (%), PCT (%), and MPV (fL) with length of ICU stay fL, femtoliter; ICU, intensive care unit; MPV, mean platelet volume; PCT, plateletcrit; PDW, platelet distribution width

Parameter	<7 (n = 57)	≥7 (n = 120)	Total	p-value	Mann-Whitney test
PDW (%) mean ± SD	20.76 ± 3.96	21.78 ± 4.6	21.45 ± 4.42	0.186	3,000.5
PCT (%) mean ± SD	0.51 ± 0.52	0.5 ± 0.52	0.5 ± 0.52	0.839	3,355.5
MPV (fL) mean ± SD	11.48 ± 1.61	11.41 ± 1.78	11.43 ± 1.72	0.829	3,351.5

**Figure 7:**
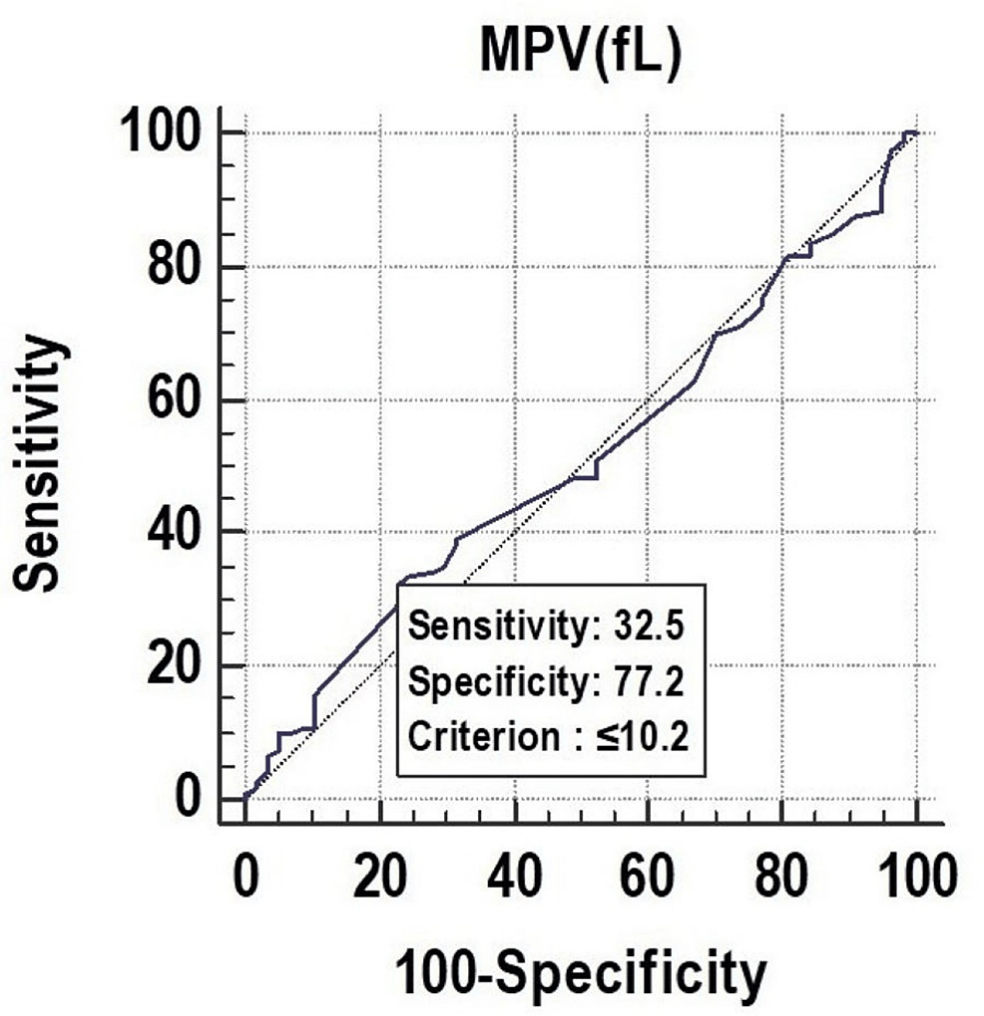
Receiver operating characteristics of MPV (fL) for predicting length of ICU stay (≥7) fL, femtoliter; ICU, intensive care unit; MPV, mean platelet volume

**Figure 8:**
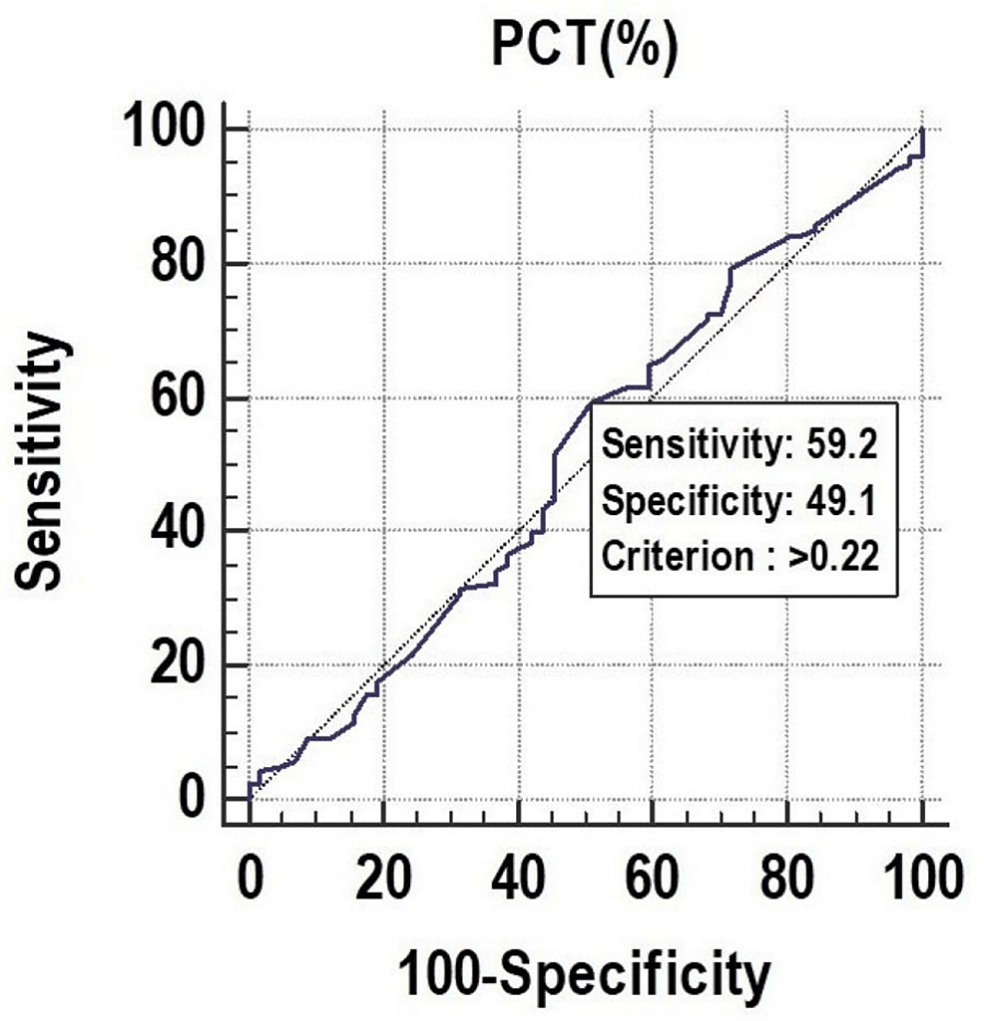
Receiver operating characteristics of PCT (%) for predicting length of ICU stay (≥7) ICU, intensive care unit; PCT, plateletcrit

**Figure 9:**
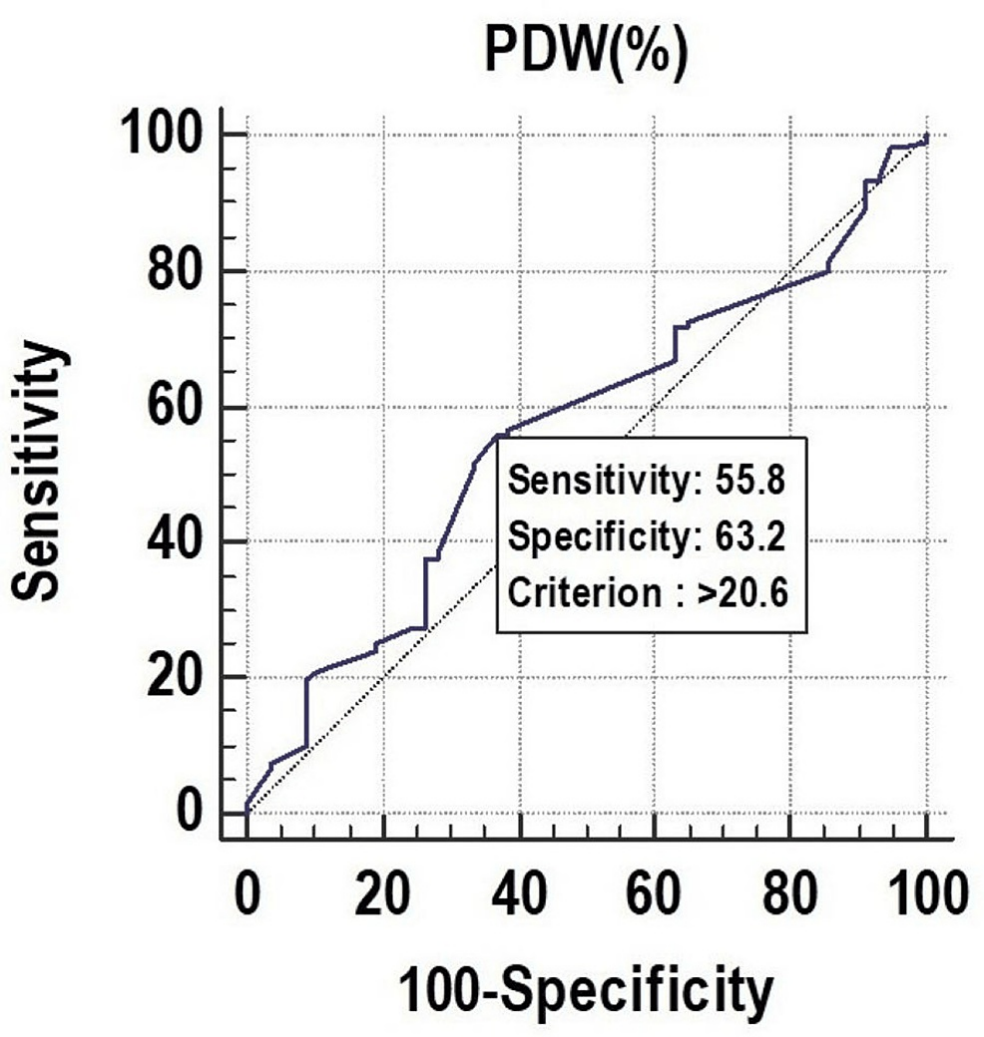
Receiver operating characteristics of PDW (%) for predicting length of ICU stay (≥7) ICU, intensive care unit; PDW, platelet distribution width

PCT had a sensitivity of 59.17%, followed by PDW (55.83%) and MPV (32.50%). In the prediction of length of ICU stay ≥7 days, MPV had the lowest sensitivity of 32.50%. On the other hand, MPV (fL) had a specificity of 77.19%, followed by PDW (%) (63.16%), and PCT (%) (49.12%). The highest positive predictive value was found in PDW (%) (76.10%), and the highest negative predictive value was found in PDW (%) (40.40%), as shown in Figure 7, Figure 8, and Figure 9.

**Figure 10:**
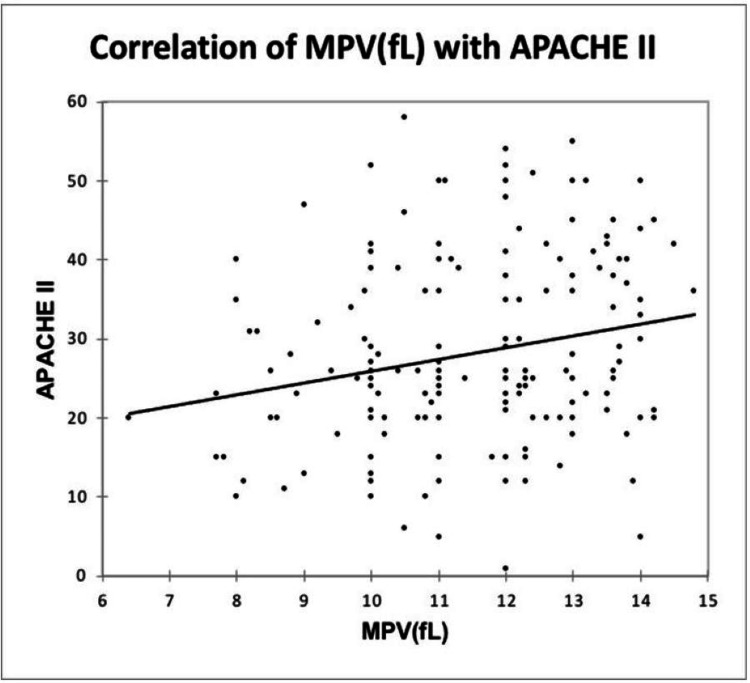
Correlation of MPV (fL) with APACHE II APACHE, Acute Physiology and Chronic Health Evaluation; fL, femtoliter; MPV, mean platelet volume

**Figure 11:**
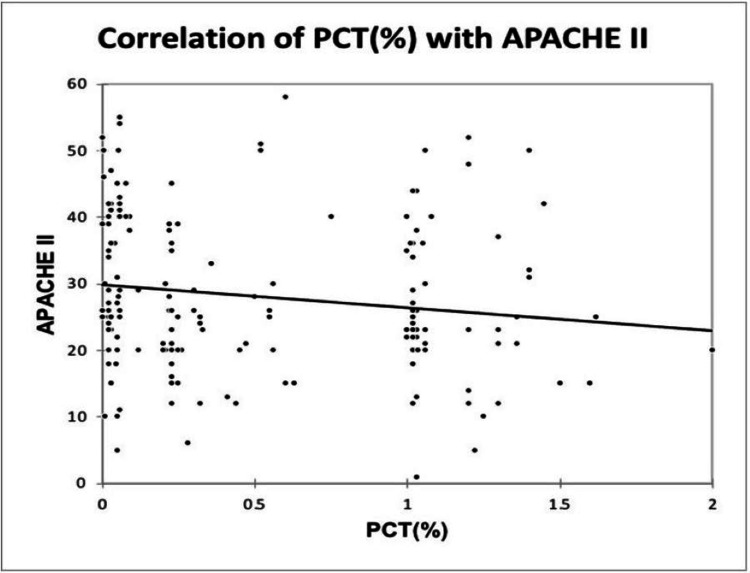
Correlation of PCT (%) with APACHE II APACHE, Acute Physiology and Chronic Health Evaluation; PCT, plateletcrit

**Figure 12:**
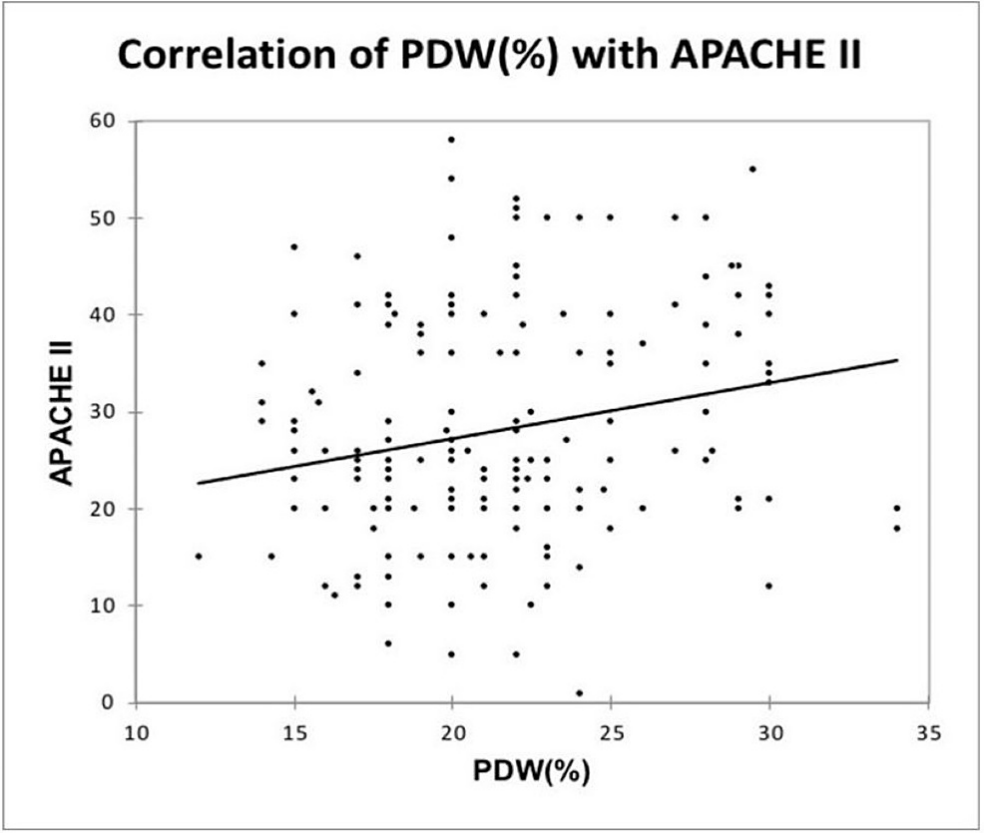
Correlation of PDW (%) with APACHE II APACHE, Acute Physiology and Chronic Health Evaluation; PDW, platelet distribution width

A significant positive correlation was seen between MPV (fL) and APACHE II, with a correlation coefficient of 0.215. A significant negative correlation was seen between PCT (%) and APACHE II, with a correlation coefficient of -0.189. A significant positive correlation was seen between PDW (%) and APACHE II with a correlation coefficient of 0.197, as shown in Figure 10, Figure 11, and Figure 12.

The variable PCT (%) was not normally distributed. Thus, a nonparametric test was used for the association. A significant association was seen in PCT (%) with Gram-positive, Gram-negative, or others (p < 0.05). The median (IQR) of PCT (%) in others was 1.4 (1.21-1.51), and Gram-positive was 0.88 (0.243-1.028), which was significantly higher as compared to Gram-negative (0.2 (0.05-0.89)). The box and whisker plot depicts the distribution of PCT (%) in the three groups, as shown in Table 5.

**Table 5:** Association of PCT (%) with Gram-positive, Gram-negative, or others PCT, plateletcrit

PCT (%)	Gram-negative (n = 50)	Gram-positive (n = 18)	Others (n = 3)	Total	p-value	Test performed
Mean ± SD	0.41 ± 0.51	0.7 ± 0.48	1.35 ± 0.3	0.52 ± 0.54	0.008	Kruskal-Wallis test; chi-squared test = 9.559
Median (IQR)	0.2 (0.05-0.89)	0.88 (0.243-1.028)	1.4 (1.21-1.51)	0.25 (0.055-1.02)		
Range	0-2	0.02-1.45	1.02-1.62	0-2		

## Discussion

In this study, there was a correlation between platelet indices on morbidity, ICU stay, mortality, and the cause of sepsis (Gram-positive or Gram-negative infections). In the present study, the platelet indices assessed were MPV, PCT, and PDW, whose mean values were 11.43 ± 1.72, 0.5 ± 0.52, and 21.45 ± 4.42, respectively. It signified that MPV and PDW increased and PCT decreased as the severity of sepsis increased.

Our findings were in line with the study by Zhang et al., who reported that patients with more severe illnesses had higher MPV values and PDW [13]. In another study, Poonam et al. also observed that among the platelet indices, PDW showed a significant positive correlation with the APACHE II score in critically ill patients with sepsis [14]. Similarly, in the study by Guclu et al., 68 patients with severe sepsis had a lower platelet count (PCT), a higher MPV, and an increased PDW [15].

The underlying mechanism for changes in the platelet indices with the increasing severity of sepsis can be explained based on platelet activation and destruction in sepsis. As platelet volume is related to platelet function and activation, platelets shift their discoid shape to a spherical shape during activation, along with the formation of pseudopodia, thereby influencing the PDW to obtain a larger surface area during platelet activation in severe sepsis [16].

In addition, platelet production increases as young platelets are also functionally more active than older platelets. The whole process overall leads to an increase in MPV and PDW. There is mild or mild to severe thrombocytopenia in sepsis, leading to low PCT. All three correlations with increasing severity of sepsis were observed in the present study, signifying the fact that platelet indices can be used as markers for assessing the severity of sepsis [17].

Among all the parameters, PCT (%) was the best predictor of mortality at a cutoff point of ≤0.22, with a 67.30% chance of correctly predicting mortality. The prediction of mortality for all the platelet indices was significant (p < 0.05).

In the study by Gao et al., as compared to the survivor group, the non-survivor group had significantly higher MPV (11.2 vs 10.3, p = 0.01), but PDW and PCT were comparable among the two groups [18].

In one of the previous studies by Orak et al., a comparison of the MPV, PDW, PLR, and platelet values of the deceased and survivors exhibited that the MPV, PDW, and PLR values of the deceased patients were high, and their platelet counts were lower than those of the survivors [19]. The difference was statistically significant in favor of mortality (p = 0.006, p < 0.001, p < 0.001, and p = 0.008, respectively). In a study conducted by Mangalesh et al., it was found that both PDW and MPV were independent predictors of mortality in sepsis and showed significant changes until Day 3 of admission [20].

In the present study, PDW showed a significant association with growth in culture, as patients with growth had significantly higher PDW as compared to those who did not have growth (22.4 ± 4.47 vs 20.81 ± 4.29, p = 0.011). Specifically, for the organisms, a significant association was seen in PCT (%) with Gram-positive, Gram-negative, or others (p < 0.05). The median (IQR) of PCT (%) in others was 1.4 (1.21-1.51), and Gram-positive was 0.88 (0.243-1.028), which was significantly higher as compared to Gram-negative (0.2 (0.05-0.89)).

The findings were in line with the Zhang et al. study, which concentrated on markers (PCT, PDW, and RDW) that can be used for the rapid diagnosis of sepsis [13]. In their study, PCT was found to be a better marker of inflammation, with the highest accuracy. The study results showed that the high PDW values found at the onset of inflammation were significantly correlated with bacteremia.

Leli et al., in a study of 571 patients, discovered that predicting bacteremia in patients with suspected sepsis using the optimal cutoff value of > 0.5 ng/ml for PCT resulted in 94% and 64%, respectively, of sensitivity and specificity [21].

Limitation

The present study is a single-center study carried out with a limited sample size, therefore limiting the participation from the rest of the Indian regions. The immature platelet fraction was not taken into account in this study. A combined list of parameters with ventilators and mortality should have been evaluated for an in-depth study. Further multicenter studies with larger sample sizes can help validate the results of the present study.

## Conclusions

Platelet indices are routinely prescribed blood parameters available even at the primary care physician level in the rural population. There was evidence of an association between platelet indices on morbidity, ICU stay, and mortality. The MPV and PDW were positively correlated with the severity of sepsis. Among all the parameters, PCT was the best predictor of the need for an invasive mechanical ventilator at a cutoff point of ≤0.22 with a 60.90% chance, as well as of mortality at a cutoff point of ≤0.22 with a 67.30% chance. Platelet indices may be associated with the cause of sepsis due to Gram-positive or Gram-negative bacterial infections. Centers with limited resources may benefit from using these readily available, cost-effective parameters to predict the severity and mortality of sepsis. This will enable practicing clinicians to provide better critical care in the ICUs as well as in predicting mortality and morbidity.
